# A D-enantiomer of the antimicrobial peptide GL13K evades antimicrobial resistance in the Gram positive bacteria *Enterococcus faecalis* and *Streptococcus gordonii*

**DOI:** 10.1371/journal.pone.0194900

**Published:** 2018-03-22

**Authors:** Helmut Hirt, Jeffrey W. Hall, Elliot Larson, Sven-Ulrik Gorr

**Affiliations:** Department of Diagnostic and Biological Sciences, University of Minnesota School of Dentistry, Minneapolis, Minnesota, United States of America; Oregon Health & Science University, UNITED STATES

## Abstract

Antimicrobial peptides represent an alternative to traditional antibiotics that may be less susceptible to bacterial resistance mechanisms by directly attacking the bacterial cell membrane. However, bacteria have a variety of defense mechanisms that can prevent cationic antimicrobial peptides from reaching the cell membrane. The L- and D-enantiomers of the antimicrobial peptide GL13K were tested against the Gram-positive bacteria *Enterococcus faecalis* and *Streptococcus gordonii* to understand the role of bacterial proteases and cell wall modifications in bacterial resistance. GL13K was derived from the human salivary protein BPIFA2. Minimal inhibitory concentrations were determined by broth dilution and a serial assay used to determine bacterial resistance. Peptide degradation was determined in a bioassay utilizing a luminescent strain of *Pseudomonas aeruginosa* to detect peptide activity. Autolysis and D-alanylation-deficient strains of *E*. *faecalis* and *S*. *gordonii* were tested in autolysis assays and peptide activity assays. *E*. *faecalis* protease inactivated L-GL13K but not D-GL13K, whereas autolysis did not affect peptide activity. Indeed, the D-enantiomer appeared to kill the bacteria prior to initiation of autolysis. D-alanylation mutants were killed by L-GL13K whereas this modification did not affect killing by D-GL13K. The mutants regained resistance to L-GL13K whereas bacteria did not gain resistance to D-GL13K after repeated treatment with the peptides. D-alanylation affected the hydrophobicity of bacterial cells but hydrophobicity alone did not affect GL13K activity. D-GL13K evades two resistance mechanisms in Gram-positive bacteria without giving rise to substantial new resistance. D-GL13K exhibits attractive properties for further antibiotic development.

## Introduction

Antimicrobial peptides (AMPs) have been investigated for several decades in an effort to develop alternatives to traditional antibiotics, which face increasing levels of bacterial resistance [1–5]. Naturally occurring AMPs are found in vertebrates, invertebrates, plants and microbes and may act on the bacterial membranes, cell wall or interior targets [6–8]. It has been proposed that the interaction of AMPs with the bacterial cell membrane is associated with relatively low probability of bacterial resistance [9], although bacteria that inhabit the host microbiome and some invading bacteria clearly have the ability to co-exist with or overcome host AMPs. Indeed, experimental resistance to AMPs has been observed [10] and the corresponding bacterial defense mechanisms could also protect against therapeutic AMPs [7, 11]. Conversely, wide-spread use of therapeutic AMPs could lead to resistance also against endogenous host-defense peptides (“arming the enemy”) and render the host unprotected against invading bacteria [12]. To address these concerns, a better understanding of the mechanisms of action and bacterial resistance to AMPs is needed [13].

We have developed the antimicrobial peptide GL13K (GKIIKLKASLKLL-NH_2_), which is derived from the human salivary protein BPIFA2 (BPI-fold family A, member 2; formerly: Parotid Secretory Protein, PSP, SPLUNC2, C20orf70) [14, 15]. L-GL13K is bactericidal and effective against Gram-negative bacteria [14], although it is susceptible to bacterial proteases [16]. L-GL13K also kills established biofilms of *Pseudomonas aeruginosa* and contributes to their eradication in combination with the aminoglycoside tobramycin [16]. Studies with L-GL13K on artificial membranes suggested selectivity for negatively charged model bacterial membranes leading to peptide-induced micellization and transient pore formation. Both mechanisms are thought to create holes that would lead to rapid cell death by loss of membrane potential and ions from the bacterial cell [17, 18].

Immobilized L-GL13K is active against the Gram-positive bacteria *Streptococcus gordonii*, causing cell rupture, reminiscent of autolysis, under continuous nutrient flow conditions [19]. To test if the soluble peptide is effective against Gram positive bacteria, the original L-amino acid version of GL13K [14] and a protease-resistant all-D-amino acid version D-GL13K [16] were tested against *Enterococcus faecalis* and *S*. *gordonii*. The enantiomers showed significant differences in their effects, suggesting that D-GL13K kills bacteria by evading bacterial resistance without causing new resistance.

## Materials and methods

### Bacterial strains and culture conditions

*E*. *faecalis* strains OG1RF (wild-type); a D-alanylation mutant (*dltA*) of OG1RF (TX5427); a protease negative, autolysis impaired mutant (*gelE*^-^, SprE^-^) of OG1RF (TX5128); vancomycin-resistant *E*. *faecalis* V583 and *S*. *gordonii* DL1 (wild-type) have previously been described [20–24]. Bacterial cultures were inoculated from frozen stock cultures and grown in Todd-Hewitt Broth (THB) (Difco, Franklin Lakes, NJ) at 37°C as stand cultures. Bacteria were cultured overnight unless otherwise mentioned. Todd-Hewitt Agar (THA) for enumeration of CFU contained 1.5% agar (Difco) in THB.

### Construction of *Streptococcus gordonii* autolysis mutant (*atlS*) and D-alanylation mutant (*dltA)*

The *S*. *gordonii* DL1 *atlS* (SGO_2013) mutant was constructed by adapting the marker-less mutagenesis system developed for *Streptococcus mutans* by Xie et al. [25]. Briefly, the *S*. *gordonii ldh* promoter and mutated *pheS* gene (pheS*) and *ermAM* antibiotic resistance marker were synthesized and cloned into pUC57 (Genscript, Piscataway, NJ). The *S*. gordonii pheS* gene contains an A316G mutation and silent mutations after codon 316 to prevent recombination at the chromosomally encoded *pheS* gene, resulting in the final plasmid, pJHMD1 (Hall et al., submitted). This approach avoids off target effects and effects of antibiotics are null. The mutants retain all 5’ and 3’ transcriptional regulators.

A markerless *atlS* in-frame deletion strain was constructed by a two-step transformation protocol. The linear JHMD1 mutagenesis cassette was PCR amplified from pJHMD1 using the JHMD1For/JHMD1Rev primer pair (Table 1). Further, approximate 0.5 kb upstream and downstream regions overlapping the start and stop codons of *atlS* were PCR amplified using primers containing complementary DNA sequences to JHMD1, atlS-upFor/atlS-upRev-JHMD1 and atlS-dnFor-JHMD1/atlS-dnRev. Each PCR product was purified, mixed in equimolar concentrations, subjected to Splice-Overlap Extension PCR (SOE-PCR) to generate a 3.1 kb atlSup-JHMD1-atlSdown PCR product, transformed into *S*. *gordonii* DL1, plated on THA containing 5 mg/L erythromycin and incubated anaerobically at 37°C for 48 hours. For the second transformation, two primer pairs, atlS-upFor/atlSupRev and atlS-dnFor/atlS-dnRev, were used to amplify the same 0.5 kb upstream and downstream regions. The upstream product contains a complementary sequence to the downstream product. The PCR products were purified and mixed in equimolar concentrations for SOE-PCR. The 1 kb product was transformed into DL1:atlSup-JHMD1-atlSdn, plated on THA containing *p*-Cl-Phe and incubated anaerobically at 37°C for 48 hours. The mutation was verified by diagnostic colony PCR. The phenotype of a *S*. *gordonii atlS* mutant has previously been described [26].

**Table 1:** Primers used in the construction of the *S*. *gordoni*i DL1 *atlS* mutant.

atlS-upFor	5'- GCT AAG CCC TGT CTG GGC TTT TTG -3'
atlS-upRev-JHMD1	5'- CTA TGC TAT GAG TGT TAT CGT TTC TCG CTT CTT TTT CAT GTA ACT CCC TCT TTA ACA C -3'
atlS-dnFor-JHMD1	5'- GTT AT CTA TTA TTT AAC GGG AGG AAA TAA CGA GGA TTT GCA AGA CCA CGT TAT CAA TAA -3'
atlS-dnRev	5'- GAA GCA TTT GCT TGA GAC GAT ACT TGA C -3'
atlS-upRev-SOE KO	5'- TTA TTG ATA ACG TGG TCT TGC AAA TCC TCG CTT CTT TTT CAT GTA ACT CCC TCT TTA ACA C -3'
atlS-dnFor SOE KO	5'- CGA GGA TTT GCA AGA CCA CGT TAT CAA TAA -3'
JHMD1-For	5’- CGA GAA ACG ATA ACA CTC ATA GCA TAG -3’
JHMD1-Rev	5’- TTA TTT CCT CCC GTT AAA TAA TAG ATA AC -3’

Primer pairs used for creating the markerless *atlS* in-frame deletion strain of *S*. *gordoni*i DL1.

The construction of the DL1 *dltA* mutant followed the equivalent protocol with the primers listed in Table 2.

**Table 2:** Primers used in the construction of the *S*. *gordoni*i DL1 *dltA* mutant.

dltA-upFor	5'- GGC TAA CAG TTT AAT GGT CTG ACT G-3'
dltA-upRev-JHMD1	5'- CTA TGC TAT GAG TGT TAT CGT TTC TCG ATT CGT CAC GAA AGG ATA CCT CTT TTA ATC -3'
dltA-dnFor-JHMD1	5'- GTT ATC TAT TAT TTA ACG GGA GG AAA TAA ATG ATG GAA ATT TTA AAA CAA CTT CCT CAC -3'
dltA-dnRev	5'- GGC ATA AAG AGC ATG AAG CGC-3'
dltA-upRev-SOE KO	5'- ATT CGT CAC GAA AGG ATA CCT CTT TTA ATC -3'
dltA-dnFor SOE KO	5'- GAT TAA AAG AGG TAT CCT TTC GTG ACG AAT ATG ATG GAA ATT TTA AAA CAA CTT CCT CAC-3'
JHMD1-For	5’- CGA GAA ACG ATA ACA CTC ATA GCA TAG -3’
JHMD1-Rev	5’- TTA TTT CCT CCC GTT AAA TAA TAG ATA AC -3’

Primer pairs used for creating the *dltA* mutant strain of *S*. *gordonii* DL1. See Methods for details.

### Peptide synthesis and handling

L-GL13K (Gly-Lys-Ile-Ile-Lys-Leu-Lys-Ala-Ser-Leu-Lys-Leu-Leu-NH_2_) [14] and D-GL13K [16] were purchased from AAPPTec (Louisville, KY) or Bachem (Torrance, CA). The lyophilized powder was stored at -20°C and aliquots were resuspended as stock in sterile 0.01% acetic acid at a concentration of 10 mg/ml and stored at 4°C. The peptides were subjected to further quality control by LC/MS analysis (College of Pharmacy, University of Minnesota) to ensure equal concentration after resuspension. Peptides were either used directly from stock or further diluted to a 1 mg/ml working stock as needed.

### Minimal inhibitory concentration

The minimal inhibitory concentration (MIC) assays were performed essentially as described for cationic AMPs [27], but using streptococcal growth-friendly THB without the addition of blood or serum components. A working stock of 1-5x10^5^ CFU/ml in 90 or 100 μl THB was added to 10 or 20 μl of a 2-fold serial peptide dilution in 0.01% acetic acid and incubated for 20 h at 37°C in 96-well polypropylene plates. The OD at 600 nm (OD_600_) was determined in a Synergy HT plate reader (BioTek, Winooski, VT). Control wells for growth without peptide and growth medium without bacteria were included. Bacterial cell numbers in the inoculum were verified by colony count.

### Analysis of protease activity

Aliquots (5 μl) of overnight cultures of *E*. *faecalis* OG1RF or TX5128 were spotted on THA containing 3% gelatin. The plates were incubated at 37°C overnight and gelatinase activity was detected as a turbid halo surrounding the bacterial colonies.

To test proteolytic processing of GL13K peptides, overnight bacterial cultures were centrifuged (5 min, 13,000 g) and the conditioned supernatants were sterile-filtered (0.22 μm, Corning). Filtered supernatant (16.8 μl) was incubated with 3.2 μl (3.2 μg) peptide at 37°C for 2 h to allow bacterial proteases in the supernatant to process the peptide. To determine residual peptide activity, 70 μl of 10 mM sodium phosphate buffer, pH 7.4 was added to each sample and then supplemented with 10 μl (10^8^ CFU) of bioluminescent *Pseudomonas aeruginosa* Xen41 (Caliper Lifesciences; now Perkin Elmer, MA) that had been washed three times in 0.9% NaCl (final maximal peptide concentration 32 mg/L). Viability of the indicator bacteria was determined after 15 min incubation at 37°C by determining bioluminescence in a Synergy HT plate reader. The luminescence detected in the absence of added peptide was used as a measure of 100% proteolysis.

### Bacterial autolysis assay

The lysis assay followed an established protocol for bacterial autolysis [28] with minor modifications. Exponentially growing cells were washed twice in PBS then washed once in ice-cold dH_2_O. The bacteria were incubated in PBS with 0.05% Triton X-100 and peptides at a concentration of 100 mg/L. OD_600_ was monitored spectrophotometrically for 180 min. In some experiments, the cells were incubated with the peptides for 2h in PBS+0.05% Triton X-100 and then cultured on THA to enumerate surviving cells.

### Bactericidal activity

These assays were performed according to our established protocol [14] with some modifications. Cells were washed three times in 0.9% NaCl and five μl of washed cells (10^9^ CFU/ml) were added to the peptides diluted 2-fold (128 to 4 mg/L) in 45 μl of 10 mM sodium-phosphate, pH 7.4. The samples were incubated at 37°C for 2 h and 10 μl were then spotted on THA plates to assess bactericidal activity.

### Biofilm assay

*E*. *faecalis* OG1RF, TX5427 or V583 were cultured in a Calgary device for 24h in THB. Biofilms were treated with peptides (100 mg/L) for 4h and surviving cells were sonicated and plated on THA for enumeration of CFUs.

### Selection for GL13K peptide resistance in *S*. *gordonii* and *E*. *faecalis*

To investigate potential resistance development against the GL13K peptides, dilutions of the peptides were prepared and incubated as described for the MIC assay. The next day (day 0), the MIC was recorded and the culture in the well with the highest peptide concentration that allowed growth (i.e 0.5 x MIC) was diluted 1:100 and used as an inoculum for the next round of selection in a new MIC assay and so forth until a stable plateau of resistance was reached [29].

### Statistical analysis

Data were analyzed as described in the figure legends, using Graphpad Prism v. 6.07 (Graphpad Software, La Jolla, CA).

## Results

### Antibacterial activity of GL13K enantiomers

In the absence of an energy source, D-GL13K effectively killed 10^8^ CFU/ml wild-type *E*. *faecalis* at 15±10 mg/L (N = 4) while 48±23 mg/L (N = 3) of L-GL13K were required for complete killing. This difference was reflected in the inhibition of bacterial growth. D-GL13K showed an average MIC of 13 mg/L, while L-GL13K did not inhibit growth of wild-type *E*. *faecalis* at concentrations up to 512 mg/L (Table 3).

**Table 3:** Minimal inhibitory concentrations of *E*. *faecalis*.

*E*. *faecalis*	L-GL13K		D-GL13K	
Strain	MIC (mg/L)	N	MIC (mg/L)	N
OG1RF	>	10	13 ± 2	13
OG1RF *dltA*	70 ± 17*	6	23 ± 6	5
TX5128(*gelE*^-^, SprE^-^)	>	5	11 ± 1	5
OG1RF *ebsG*	>	5	7 ± 1	4
OG1RF::pMSP7551	>	5	10 ± 0	5
OG1RF::pMSP7551+nisin	>	5	10 ± 0	5
V583	>	4	10 ± 0	4

Minimal inhibitory concentrations were determined as described in Methods. The MICs are shown as mean ± SEM for L-GL13K and D-GL13K. >) Mean MIC is above the tested concentration range (this value was set at 200 mg/L for data analysis). MIC dilution series were analyzed individually and statistical outliers from all series were removed using the ROUT method (Q = 1%), as provided in Graphpad Prism. The cleaned data were analyzed by non-parametric Kruskal-Wallis test and compared to the wild-type strain (OG1RF) with Dunn’s multiple comparison post-test.

*) different from OG1RF, P<0.0001.

### Effect of proteases on peptide activity

Actively growing *E*. *faecalis* produce proteases that contribute to their resistance to AMPs [30]. We have previously observed that L-GL13K is more sensitive to bacterial proteases than D-GL13K [16]. To determine if the high resistance of growing *E*. *faecalis* to L-GL13K could be due to degradation by bacterial proteases, the peptides were tested against the protease deficient strain TX5128. Surprisingly, the MICs for this strain were not different from those of the wild-type strain OG1RF (Table 3). Thus, L-GL13K did not inhibit growth of TX5128 while D-GL13K exhibited an MIC of 11 mg/L.

**Fig 1:**
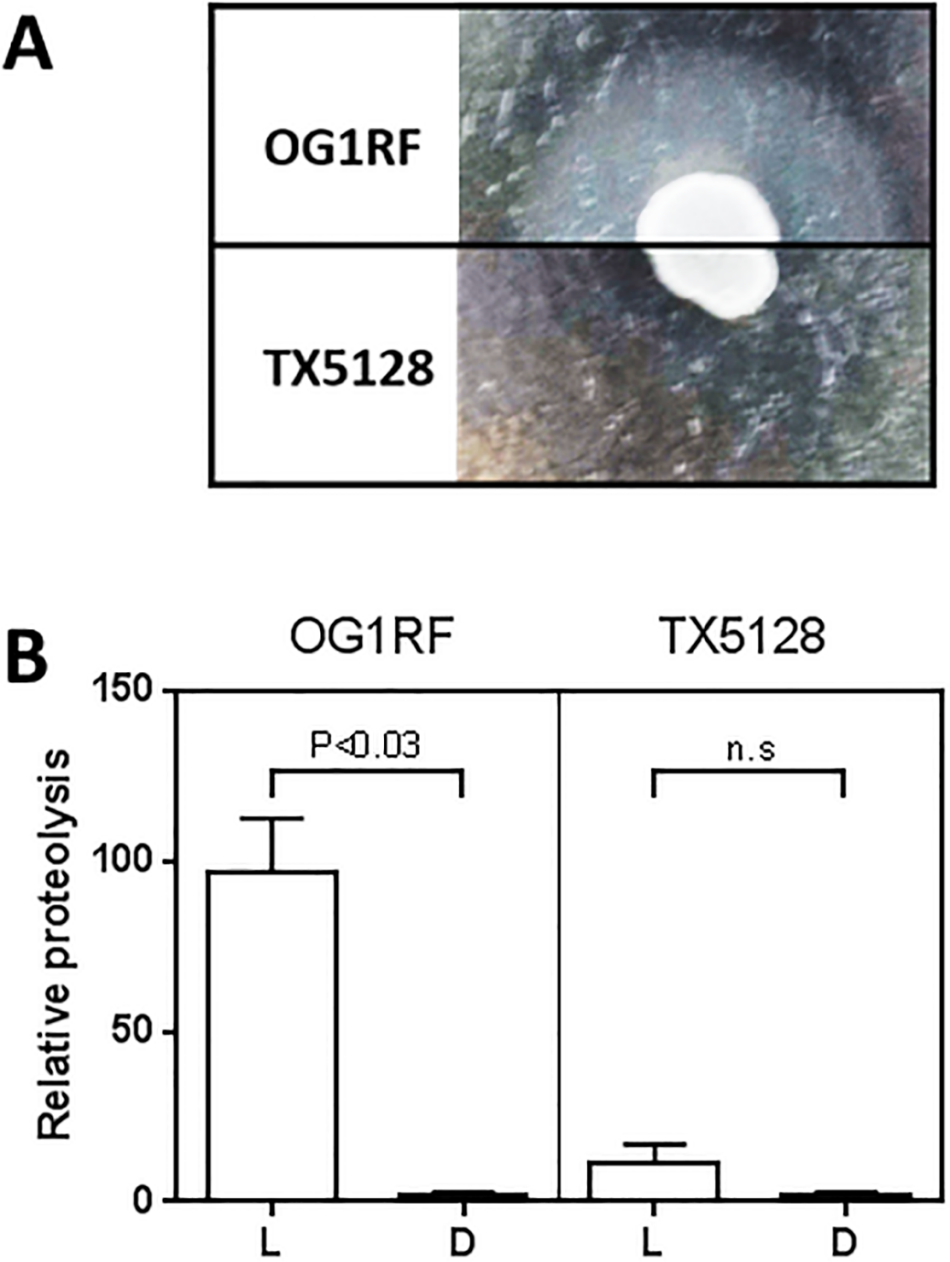
Relative proteolysis of GL13K peptides. (A): Wild-type (OG1RF) or the protease-negative strain (TX5128) of *E*. *faecalis* was cultured on THA containing 3% gelatin. Gelatinase activity was visualized as a turbid halo around the bacterial colonies; (B): L-GL13K (L) or D-GL13K (D) (32 mg/L) was incubated with conditioned supernatant of wild-type (OG1RF) or the protease-negative strain (TX5128) of *E*. *faecalis* for 2 h. Residual peptide activity was determined by adding bioluminescent *P*. *aeruginosa* Xen41 to the mixture. Bioluminescence from surviving bacteria was used as a measure of proteolytic degradation of each peptide and expressed relative to bacterial bioluminescence in the absence of peptides (equivalent to 100% proteolysis). Data are shown as mean ± SEM from three independent experiments and were analyzed by paired t-test for each strain. *n*.*s*., not significant.

The lack of gelatinase activity in TX5128 was verified by culture on gelatin agar [31], which resulted in a halo due to gelatin degradation around wild-type OG1RF but not around TX5128 (Fig 1A). To test if the *E*. *faecalis* proteases can in fact degrade L-GL13K, the peptides were incubated with conditioned culture supernatants from wild-type (OG1RF) and protease-deficient *E*. *faecalis* (TX5128) and residual peptide activity tested against *P*. *aeruginosa*, which is killed by both peptide enantiomers [16]. As expected, conditioned supernatant from wild-type *E*. *faecalis* caused almost complete proteolysis of L-GL13K but not D-GL13K. In contrast, the protease-deficient strain TX5128 did not show significant proteolysis of either peptide enantiomer (Fig 1B).

### Role of autolysis in bactericidal activity

**Fig 2:**
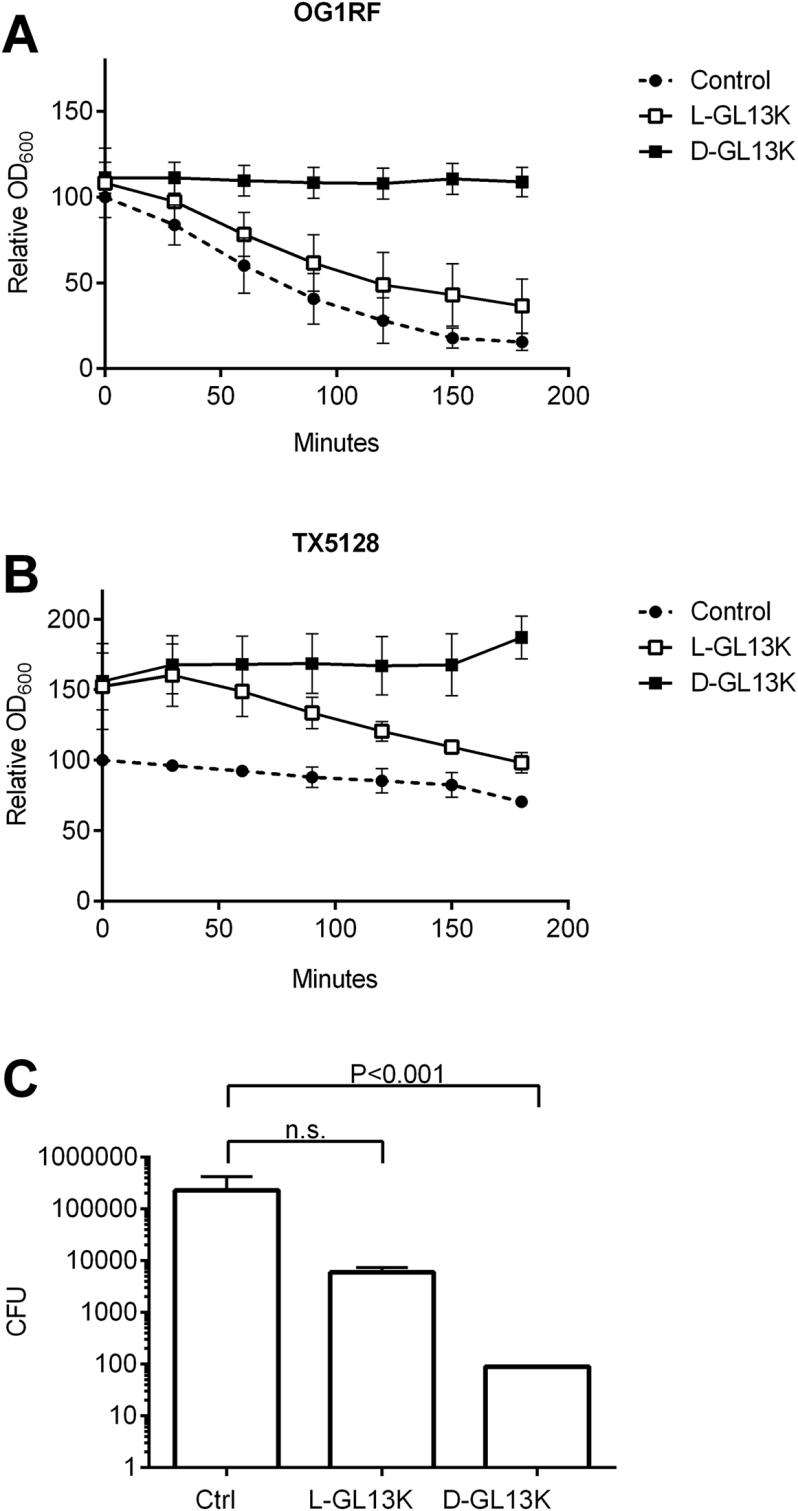
Effect of GL13K peptides on Triton X-100 induced autolysis. *E*. *faecalis* OG1RF (A) or the autolysis-deficient strain TX5128 (B) were incubated in PBS with 0.05% Triton X-100 in the absence of peptide (control) or in the presence of 100 mg/L of L-GL13K or D-GL13K (D). The OD_600_ was recorded for 180 min and expressed as a percent of starting OD_600_ of the control sample. (C): Bactericidal activity of GL13K peptides in PBS+0.05% Triton X-100. *E*. *faecalis* OG1RF were incubated with 0.05% Triton X-100 in the absence of peptide (Ctrl) or with L-GL13K or D-GL13K for 2h. Surviving CFU were enumerated and are shown as mean ± SEM (N = 5). Peptide treated samples were compared to the untreated control by Kruskal-Wallis test with Dunn’s multiple comparison post-test. *n*.*s*., not significant.

The protease-deficient TX5128 strain of *E*. *faecalis* is also deficient in autolysis [32], suggesting that autolysis is not necessary for bacterial killing by D-GL13K (Table 3). To test this, cell lysis was induced with Triton X-100 [28, 33], which caused an 80% decrease in OD_600_ after three hours in wild-type *E*. *faecalis* (Fig 2A) while the autolysis impaired mutant (TX5128) showed strongly reduced autolysis with a reduction of OD_600_ of only about 25%. L-GL13K somewhat reduced autolysis of wild-type bacteria while no autolysis was seen in the presence of D-GL13K (Fig 2A and 2B). The reduction of autolysis by the peptide enantiomers correlated with bacterial killing under autolysis conditions (Fig 2C). Thus, L-GL13K showed a small, yet non-significant, reduction of cell numbers while D-GL13K reduced viable cells by 3log10. These results suggest that D-GL13K kills *E*. *faecalis* independent of autolysis.

### Effect of D-alanylation

The cell wall of Gram-positive bacteria consists of a peptidoglycan matrix containing teichoic and lipoteichoic acids that can be D-alanylated by the *dlt*-operon, which lowers their susceptibility to cationic AMPs [34]. To test if this defense mechanism contributes to the differential effect of the GL13K enantiomers, they were tested against a *dltA* mutant of *E*. *faecalis* (TX5427) (Table 3). L-GL13K exhibited a substantially reduced MIC against the *dltA* mutant, while the activity of D-GL13K was not affected.

**Fig 3:**
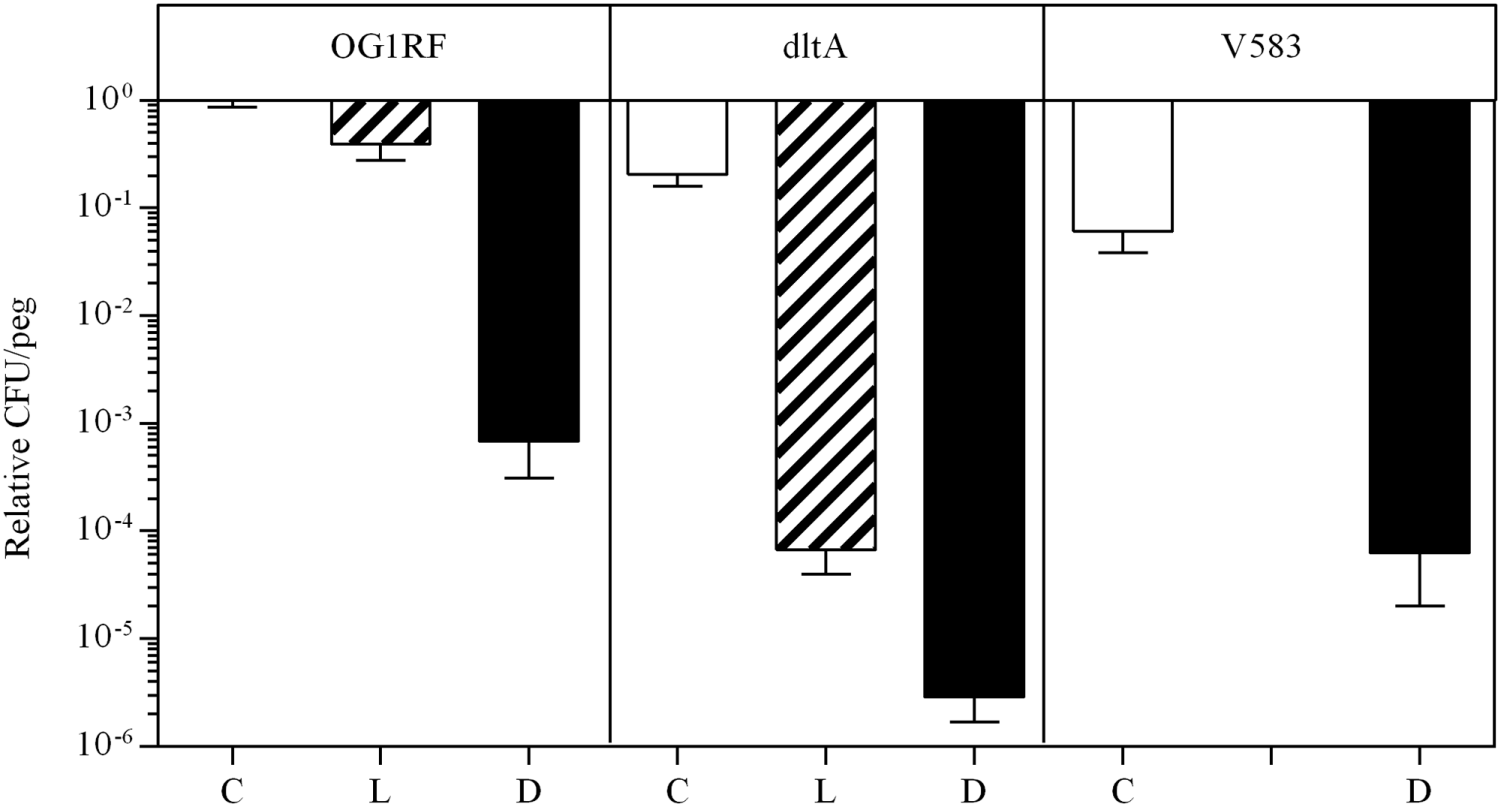
Effect of GL13K peptides on biofilms of *E*. *faecalis*. **Twenty-four hour** biofilms of OG1RF; OG1RF *dltA* or V583 were treated with 100 mg/L of L-GL13K (L) or D-GL13K (D). Surviving CFU were enumerated from each peg and expressed relative to the mean CFU recovered from pegs of wild-type cells treated with buffer alone (OG1RF—C). Data from 2–4 experiments are expressed as mean ± SEM (N = 6–13). V583 was only treated with D-GL13K.

Biofilms of *E*. *faecalis* showed similar differences in susceptibility to the GL13K enantiomers (Fig 3). L-GL13K was ineffective against wild-type biofilms but reduced viable CFU in the *dltA* mutant by three orders of magnitude, relative to the wild-type biofilm. D-GL13K was highly effective against both strains. The D-enantiomer was also tested against biofilm of a vancomycin-resistant strain of E. faecalis (V583). Although the untreated V583 biofilm contained fewer CFUs than that of the wild-type strain, D-GL13K reduced the viable biofilm CFUs by 3log10 in both strains (Fig 3). Consistent with this finding, the MICs for D-GL13K were similar for wild-type and vancomycin-resistant *E*. *faecalis* (Table 3). Thus, the relative resistance of *E*. *faecalis* to L-GL13K appears to depend partially on D-alanylation, while D-GL13K is not affected by this defense mechanism or vancomycin resistance.

**Fig 4:**
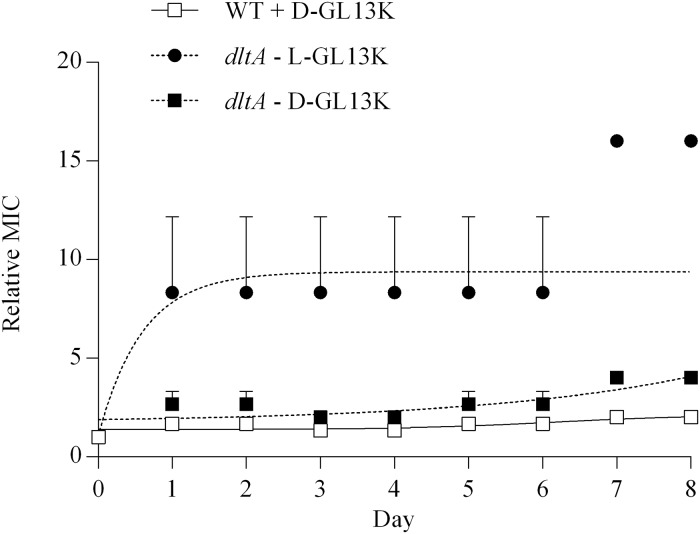
Development of resistance in *E*. *faecalis*. MICs for L-GL13K (circles) and D-GL13K (squares) were recorded on consecutive days using *E*. *faecalis* OG1RF (wild-type) (open symbols) (D-enantiomer only) and *dlt*A (closed symbols). The MIC for each day is expressed relative to the starting MIC (day 0). Data from three experiments are shown as mean ± SEM.

To test if *E*. *faecalis* TX5427 can regain resistance to GL13K peptides, serial MIC assays were performed in the presence of L-GL13K or D-GL13K. L-GL13K increased the MIC of the *dltA* mutant 8-fold after two rounds of selection, while the D-enantiomer caused only a 2-fold increase of the MIC after seven rounds (Fig 4). Similarly, D-GL13K did not substantially increase the MIC of wild-type *E*. *faecalis*. Thus, the *dltA* mutant (TX5427) appears to regain resistance to L-GL13K while remaining highly susceptible to D-GL13K.

### Surface hydrophobicity of bacterial strains

**Fig 5:**
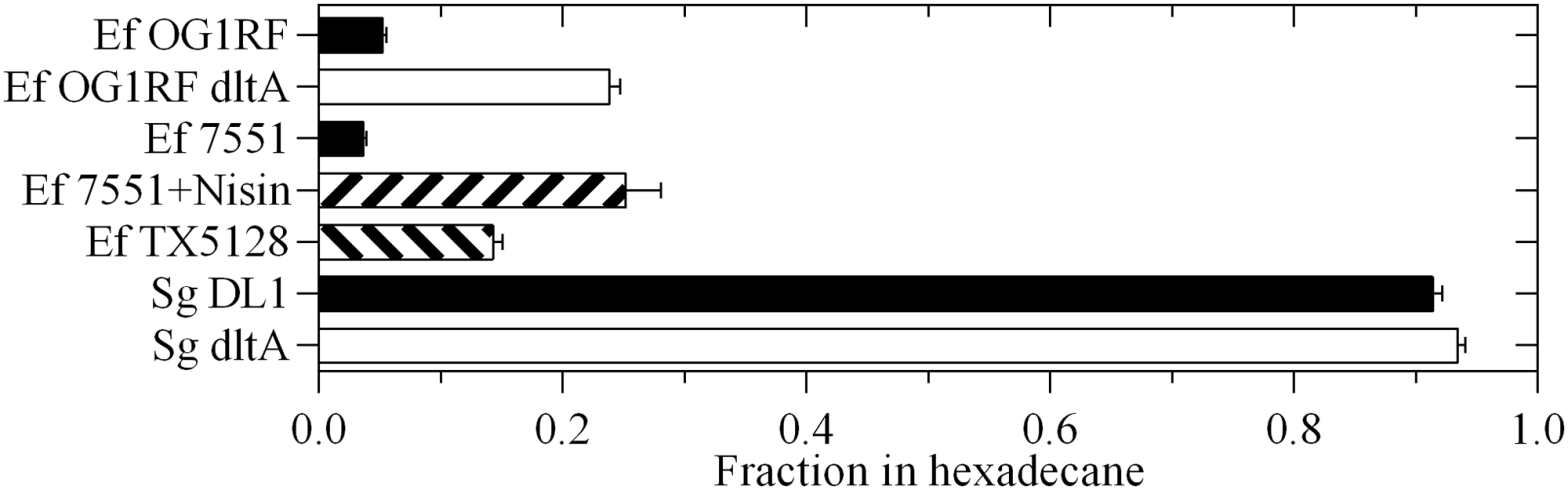
Surface hydrophobicity of bacterial strains. Bacteria were fractionated into hexadecane as a measure of surface hydrophobicity. Ef–*E*. *faecalis*; Sg–*S*. *gordonii*. 7551—OG1RF::pMSP7551. Data from 2–3 experiments are shown as mean ± SEM (N = 6–9).

Lipoteichoic acid has been correlated with surface hydrophobicity of streptococci [35]. D-alanylation of teichoic acids protects bacteria from cationic peptides by masking negative charges in the cell wall, surprisingly this does not affect cell surface hydrophobicity in *Lactococcus lactis* [36]. To determine if D-alanylation affected cell surface hydrophobicity of *E*. *faecalis*, wild-type and mutant bacteria were tested for adhesion to hydrocarbon. Wild-type OG1RF were highly hydrophilic with only 5% adhering to hexadecane (Fig 5). The *dltA* mutant showed 5-fold higher hydrophobicity. Since this difference in hydrophobicity correlated with the different susceptibility to L-GL13K, we further tested this correlation with a mutant that overexpresses the hydrophobic cell surface protein EbsG in response to induction by nisin (OG1RF::pMSP7551) [37]. Un-induced cells showed 4% hydrophobicity, i.e. similar to wild-type cells, and this increased to 25% in nisin-induced cells, i.e. similar to the *dltA* mutant (Fig 5). Despite this difference, both non-induced and induced cells were resistant to L-GL13K while their sensitivity to D-GL13K was similar to that of wild-type OG1RF cells (Table 3). These results suggest that changes in D-alanylation but not surface hydrophobicity are correlated with bacterial resistance to L-GL13K.

### Peptide effects on *Streptococcus gordonii*

To determine if the role of D-alanylation in peptide resistance was specific for *E*. *faecalis*, the related oral commensal bacteria *S*. *gordonii* were tested. *S*. *gordonii* are highly hydrophobic with over 90% binding to hexadecane (Fig 5). A *dltA* mutant of these bacteria may also exhibit increased hydrophobicity although the increase is only about 2% (P<0.06). Consistent with the results for *E*. *faecalis*, wild-type *S*. *gordonii* are resistant to L-GL13K while a *dltA* mutant shows an MIC of 11 mg/L to L-GL13K (Table 4). D-GL13K inhibits the growth of both wild-type and *dltA* mutant bacteria with MICs of 4–5 mg/L (Table 4). Thus, resistance of *S*. *gordonii* to the GL13K enantiomers is also correlated with D-alanylation but not surface hydrophobicity.

**Table 4:** Minimal inhibitory concentrations of *S*. *gordonii*.

*S*. *gordonii*	L-GL13K		D-GL13K	
Strain	MIC (mg/L)	N	MIC (mg/L)	N
DL1	>	7	5 ± 0.6	7
DL1 *dltA*	11 ± 3*	3	4 ± 2	3
DL1 *atlS*	63 ± 8	6	4 ± 1	4

Minimal inhibitory concentrations were determined as described in Methods. The MICs are shown as mean ± SEM for L-GL13K and D-GL13K. >) Mean MIC is above the tested concentration range (this value was set at 200 mg/L for data analysis). MIC dilution series were analyzed individually and statistical outliers from all series were removed using the ROUT method (Q = 1%), as provided in Graphpad Prism. The cleaned data were analyzed by non-parametric Kruskal-Wallis test and compared to the wild-type strain (DL1) with Dunn’s multiple comparison post-test.

*) different from DL1, P<0.006.

**Fig 6:**
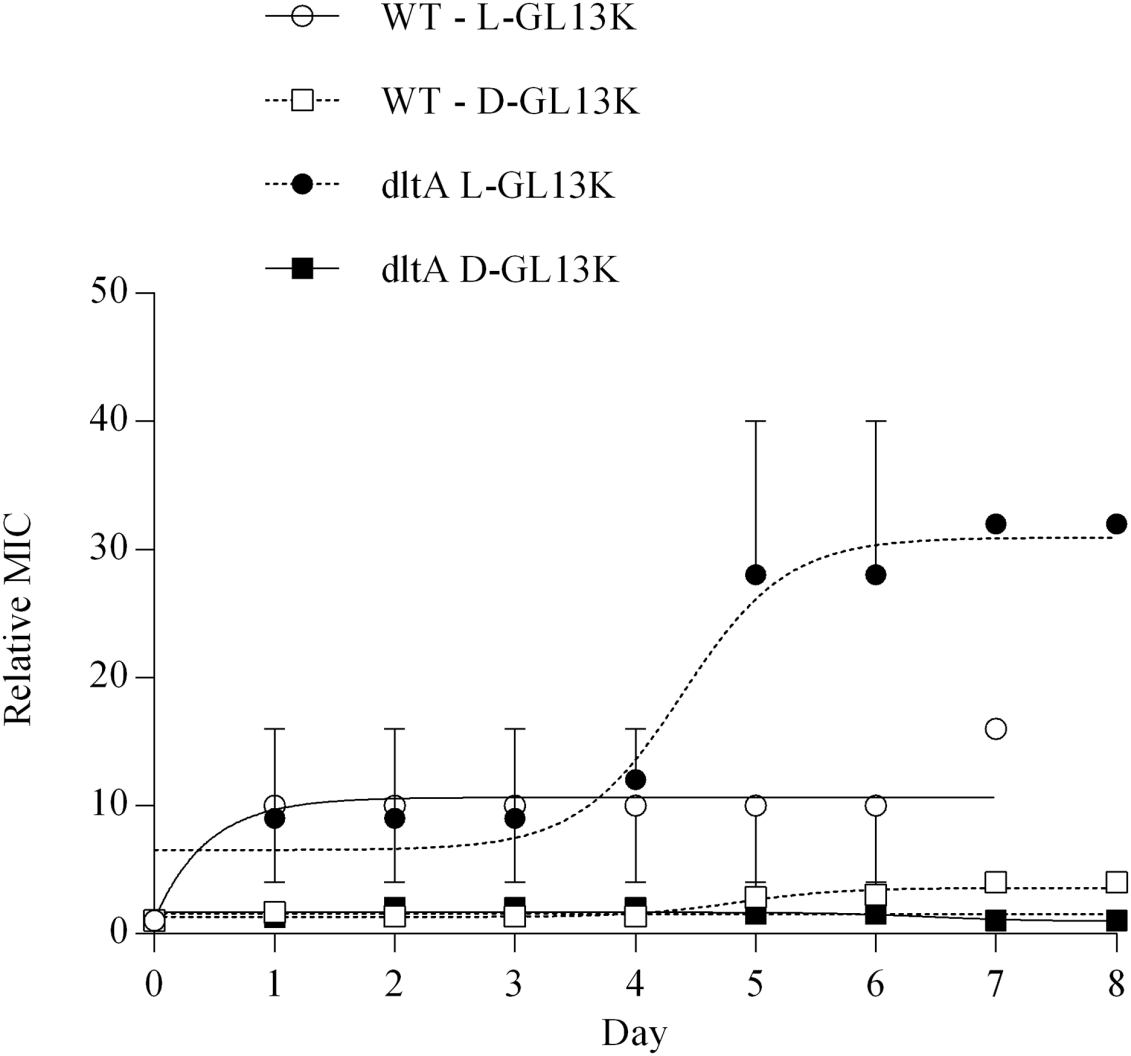
Development of resistance in *S*. *gordonii*. MICs for L-GL13K (circles) and D-GL13K (squares) were recorded on consecutive days using *S*. *gordonii* DL1 (wild-type) (open symbols) (D-enantiomer only) and *dlt*A (closed symbols). The MIC for each day is expressed relative to the starting MIC (day 0). Data from 2–3 experiments are shown as mean ± range.

As in *E*. *faecalis*, repeated treatment of *S*. *gordonii* with sub-inhibitory concentrations of D-GL13K caused little change of the MIC of wild-type and *dltA* bacteria, while L-GL13K caused a substantial increase of the MIC reaching 6-fold in wild-type bacteria and 16-fold in the *dltA* mutant (Fig 6). An autolysin-deficient mutant of *S*. *gordonii* was effectively inhibited by D-GL13K (Table 4), confirming that autolysis is not required for antibacterial activity.

## Discussion

Antimicrobial peptides have been proposed as an alternative to traditional antibiotics [13, 38, 39] and new peptides and derivatives are continuously generated and investigated [40, 41]. It has been suggested that these peptides would be less susceptible to bacterial resistance mechanisms since they directly attack the bacterial cell membrane [9]. On the other hand, resistance can develop [10] and concerns have been raised that bacterial resistance to therapeutic peptides could also render bacteria resistant to the host’s defense peptides (arming the enemy) [12]. Indeed, it is increasingly clear that bacteria have a variety of defense mechanisms that can prevent cationic antimicrobial peptides from reaching the cell membrane, including secreted proteases or modification of the cell surface, i.e. the cell wall in Gram-positive bacteria or the outer membrane in Gram-negative bacteria [4, 11, 13, 42].

Secreted proteases are effective defense mechanisms against unmodified peptides. Indeed, the protease-resistant D-GL13K is much more effective against *P*. *aeruginosa* [16], *S*. *gordonii* and *E*. *faecalis* (this report) than the protease-susceptible L-enantiomer of the peptide. Similarly, a difference in MIC between the L- and D-enantiomers was reported for the synthetic peptide IK8 [43], whereas the MIC did not differ between the L- and D-enantiomers of the antimicrobial peptide anoplin [44]. Many antimicrobial peptides are resistant to host and bacterial proteases and D-amino acids are commonly found in these peptides [45]. However, it appears that protease-resistance is not the only difference between L- and D-enantiomers of AMPs. The present results with the protease negative strain *E*. *faecalis* TX5128 showed that the activity of L-GL13K also was lower than that of D-GL13K in the absence of bacterial proteases, suggesting that additional resistance mechanisms may function in these Gram-positive bacteria.

Although previous results with immobilized GL13K had suggested a role for autolysis in bacterial killing [19], the present results indicate that autolysis is not necessary for killing of *E*. *faecalis*.

A second major bacterial defense mechanism against cationic AMPs involves the reduction of the overall negative surface charge of outer membrane (Gram-negative) or cell wall (Gram-positive) components. Several Gram-negative species, including *Porphyromonas gingivalis* [46], reduce the overall negative charge of the cell surface by modifying lipopolysaccharides, thereby reducing susceptibility to AMPs [47, 48]. Gram-positive bacteria use D-alanylation of teichoic and lipoteichoic acids to reduce the negative surface charge of the cell wall and mutants that are unable to modify these molecules are consequently more sensitive to AMPs [49–51]. Indeed, L-GL13K was more effective against the *dltA* mutants of both planktonic bacteria and biofilms of *S*. *gordonii* and *E*. *faecalis* than the wild-type strains. In contrast, D-GL13K was highly effective against both mutant and wild-type bacteria. This could be relevant for potential clinical applications since Gram-positive bacteria have been shown to upregulate the *dlt*-operon to mask lipoteichoic acids with D-alanine in response to the challenge by antimicrobial peptides [52, 53]. A peptide that is not affected by D-alanylation-status may therefore retain efficacy under these conditions.

In addition to D-alanylation, the D-enantiomer of GL13K was also able to overcome the resistance mechanisms in the vancomycin resistant strain V583. The ability of the *dlt* mutant to regain resistance to L-GL13K but not D-GL13K further strengthens the clinical potential of the latter peptide.
